# Zinc finger protein 800 (*ZNF800*) promotes proliferation and migration of lower-grade glioma and is associated with immune infiltration

**DOI:** 10.1371/journal.pone.0324426

**Published:** 2025-07-11

**Authors:** Zirui Huang, Bo Pang, Tengfei Guo, Youlei Yang, Xingbo Cheng, Zhizhao Yang, Kezheng Mao, Zhendong Liu, Rongjun Qian

**Affiliations:** 1 Department of Neurosurgery, Henan University of Traditional Chinese Medicine, Zhengzhou, Henan Province, China; 2 Department of Neurosurgery, The Fourth Medical Center of Chinese PLA General Hospital, Beijing, China; 3 Department of Neurosurgery, Henan University People’s Hospital, Zhengzhou, Henan Province, China; 4 Department of Spinal Cord Surgery, Henan Provincial People’s Hospital, Zhengzhou, Henan Province, China; 5 Department of Neurosurgery, Zhengzhou University, Zhengzhou, Henan Province, China; 6 Department of Neurosurgery, Henan Provincial People’s Hospital, Zhengzhou, Henan Province, China; Queen's University Belfast, UNITED KINGDOM OF GREAT BRITAIN AND NORTHERN IRELAND

## Abstract

*ZNF800* is a novel gene affecting the malignant progression of several cancers. However, the involvement of *ZNF800* in the malignant evolution of lower-grade gliomas (LGG) and poor prognosis of patients remains unclear. This study comprehensively revealed the association between *ZNF800* and LGG malignant progression by analyzing 958 clinical samples from multiple public databases. The mRNA and protein expression levels of *ZNF800* were higher in LGG tissues than in non-LGG tissues and were associated with malignant clinical features associated with a poor prognosis. High *ZNF800* expression was closely related to the infiltration of some immune cells, particularly CD8 + T cells and dendritic cell activation. *ZNF800* was positively associated with several immune checkpoint genes, such as CD274 and PDCD1 encoding PD-1 and PD-L1, respectively. Moreover, knocking down *ZNF800* can significantly inhibit the proliferation and invasion ability of glioma cells. Therefore, *ZNF800* can be used as an effective biomarker for LGG diagnosis and prognosis and can provide a theoretical basis for potential targets of combined immunotherapy in patients with LGG.

## Introduction

Glioma, originating from neural stem or progenitor cells in the central nervous system, is the most common primary malignant tumor of the intracranial brain parenchyma, which is characterized by intracranial hypertension symptoms and neurological alterations in the area of occupancy, which poses a great challenge to the quality of life of patients with gliomas, as well as to the socioeconomic and other aspects [1]. Currently, according to the World Health Organization (WHO) 2016 classification system and The Cancer Genome Atlas (TCGA), gliomas are generally categorized into two types, i.e., Lower-grade glioma (LGG) and high-grade glioma (GBM) [2]. Although the prognosis of LGG patients is relatively better compared to GBM patients, LGG accounts for approximately 43.2% of all gliomas, with a median survival rate of 7 years, and remains a malignant disease that plagues the health and safety of human beings [3,4]. In addition to this, it has been shown that LGGs have a high recurrence rate due to the aggressive growth of LGG cells, with 70% of LGGs eventually progressing to higher grade gliomas within 5–10 years [5]. More regrettably, despite aggressive treatment measures, i.e., maximal tumor resection combined with adjuvant neoadjuvant radiotherapy and neoadjuvant chemotherapy, these therapeutic measures have only delayed the patients’ lives to a certain extent, and their treatment outcomes are far from ideal expectations [6]. The fundamental reason is that the pathogenesis of glioma has not been fully elucidated. Therefore, the discovery and clarification of more key molecules that can regulate the pathological process of LGG is not only of great practical significance to improve the understanding of the malignant evolution of LGG, but even can be used in clinical treatment in the future to provide more and better therapeutic means for LGG patients.

The development of malignant tumors is the result of the dysregulation of many processes that together contribute to the development of malignant tumors, among which the abnormal regulation of genetic material is one of the important reasons for the malignant evolution of tumors, including genomic variation, abnormal transcription, and translational imbalance [7]. An abnormal disorder of the expression spectrum of transcriptomics is necessary for malignant processes in various tumors, and the imbalance of transcription factor regulation is critical in gene expression disorder and cancer initiation and progression [8]. Zinc finger proteins are the largest family of transcriptional regulators. The family of canonical zinc finger proteins includes three classes: the leucine domain, the Krüppel-associated box, and the BTB domain (broad complex, Tramtrack, and Bric-à-brac) [9]. Zinc finger proteins possess various biological functions, such as DNA recognition, transcriptional activation, and apoptosis regulation, particularly in different tumor microenvironments that promote and inhibit tumor growth [10–12]. For example, *ZNF545* and *ZNF307* are downregulated in tumor expression and negatively control cell growth. *ZNF224*, a pathogenic gene in chronic lymphocytic leukemia and bladder cancer, plays a role in chemotherapy and apoptosis and promotes cell proliferation. *ZNF800* primarily plays a role in cell growth and is a major regulator of fat gene expression and cardiometabolic traits. However, whether *ZNF800* can promote or inhibit tumor growth remains poorly understood [13,14]. At present, there is no relevant research to reveal the role of *ZNF800* in LGG, as well as its prognostic and regulatory mechanisms for LGG patients. Therefore, in this study, we investigated whether *ZNF800* is involved in the malignant process of LGG, its effect on patient outcomes, and whether it can be used as a biotherapy target to improve the prognosis of patients with LGG.

This study explores for the first time the potential regulatory mechanism and therapeutic value of *ZNF800* in LGG. We collected and analyzed clinical information and sequencing data of LGG from numerous well-known databases, and conducted basic experiments to verify it. Based on the potential value of anti-tumor immunotherapy, we systematically analyzed the relationship between the expression of *ZNF800* and immune cell infiltration and multiple well-known immune checkpoints in the LGG immune microenvironment. Filling the gap in the biological function of *ZNF800* in the field of LGG. In summary, this study provides a theoretical basis for *ZNF800* as a potential combined immunotherapy target for LGG and has the potential to prolong patient survival.

## Materials and methods

### Data collection

Gene expression profiling interactive analysis (GEPIA; http://gepia.cancer-pku.cn/) can rapidly identify the expression level changes in target tumor genes [15]. Therefore, we first detected whether *ZNF800* had abnormal expression in LGG tissues using the GEPIA database. Based on the results of the GEPIA database, we further screened the clinical data of 503 patients with LGG with complete clinical information from TCGA database (https://portal.gdc.cancer.gov/) to verify the effect of *ZNF800* expression on the prognosis, clinical features, and immune microenvironment of patients with LGG [16]. In addition, to validate the results of the TCGA database, we downloaded 403 complete clinical information and RNA sequencing data from the Chinese Glioma Genome Atlas (CGGA; http://www.cgga.org.cn/) database for analysis.[17]. Specific clinical patient information for the two databases is shown in S1 and S2 Tables. Since proteins are the main executors of mRNAs for biological functions, we also obtained immunohistochemical information of normal brain tissues and LGG tissues from the Human Protein Atlas (HPA; https://www.proteinatlas.org/) database to analyze the protein expression levels of *ZNF800* levels.

### Patients and tissue preparation

The recruitment period for 5 samples of adult epilepsy patients and 4 samples of adult LGG patients is from June 1, 2020 to December 31, 2020. All tissue specimens were obtained from the hospital operating room and stored in a −80°C refrigerator for backup, and all patients sign a written informed consent form. This study was approved by the Ethics Committee of Henan Provincial People’s Hospital (2020107).

### Cell culture and treatment

We selected glioma cell lines SHG44, SW1088 for in vitro experiments to validate the expression pattern of *ZNF800* at the cellular level. LGG tissue samples were collected in the operating room of Hospital and immediately immersed in normal saline. Subsequently, the tissues were cut into pieces using ophthalmic scissors in the ultra-clean workbench, digested with 0.25% trypsin for 10 min, and centrifuged for 5 min. The red blood cell lysate was added for 5 min, and the cells were obtained by centrifugation, PBS cleaning, and re-centrifugation. Using a medium containing 89% high glucose (Cat PM150210; Procell, China), 10% fetal bovine serum (FBS) (Cat 10099141; Gibco, Thermo Fisher Scientific, Waltham, MA, USA), and 1% penicillin and streptomycin (Cat P1400; Solarbio, Beijing, China), the cells were cultured in a constant temperature incubator at 37 °C and 5% CO_2_. When the cells reached approximately 100% fusion, then passaging culture was performed at 50% fusion and subsequent experiments were performed.

### RNA extraction and RT-qPCR

Total RNA was collected using Total RNA Kit I (R6834-02; Omega, Norcross, GA, USA), and total RNA concentration was measured using NanoDrop (Thermo Fisher Scientific), according to the manufacturer’s instructions. Next, cDNA was synthesized on a T100 thermal cycler device (Bio-Rad Laboratories, Hercules, CA, USA) according to the instructions of NovoScript Plus All-in-one 1st Strand cDNA Synthesis SuperMix (Novoprotein, Beijing, China). The obtained cDNA was quantitatively analyzed for *ZNF800* mRNA expression on the StepOne Plus Real-Time PCR System equipment (Thermo Fisher Scientific) according to the manufacturer’s instructions of NovoStart® SYBR qPCR SuperMix Plus (Novoprotein). As an internal reference gene, *18S* was used to standardize *ZNF800* mRNA expression. The results of cell RT-qPCR were calculated using the 2^-ΔΔCT^ mode, and those of tissue RT-qPCR were calculated using the –ΔCT mode. The primer sequences for *18S* and *ZNF800* were as follows: 18S forward: 5′-GTAACCCGTTGAACCCCATT-3′; 18S reverse: 5′-CCATCCAATCGGTAGTAGCG-3′; *ZNF800* forward: 5′-TCGTGCTGTGGCTGCTCTCA-3′; *ZNF800* reverse: 5′-CGATCTCCTTCTCATGGCGG-3′.

### Cell transfection

Collect logarithmic growth phase SHG44 and SW1088 cells using trypsin digestion method, inoculate them into 6-well plates with 1 × 10^5^ cells per well, and divide them into si-NC group, si-*ZNF800*–1 group, and si-*ZNF800*–2 group. Afterwards, incubate in a culture incubator for 24 hours, and mix siRNA from each group with lipo 3000 and culture medium to add to the cells. After 6 hours of cultivation, change the culture medium to fresh and then incubate for another 48 hours. The sense of si-*ZNF800*–1 is 5’-CAGAGGAUUACCAAUUUATT-3’, and the sense of si-*ZNF800*–2 is 5’-CAACCUUCCUGAUGUAAAUTT-3’. The expression of *ZNF800* was detected by qRT-PCR and Western blot, to verify the effect of *ZNF800* siRNA transfection.

### CCK8 assay

Resuspension SHG44 and SW1088 cells from si-NC group, si-*ZNF800*–1 group, and si-*ZNF800*–2 group into single-cell suspensions. After cell counting, evenly lay the plates with a density of 1 × 10^3^ cells per well on a 96 well plate, and culture for 12 hours. After the cells adhere to the wall, set the time point to 0 hours and replace the culture medium in the 96 well plate with the prepared CCK8 (Cell Counting Kit-8) working solution (the ratio of culture medium to original solution is 10:1). After continuing to culture the cells in the incubator for 4 hours, use a multifunctional microplate detector (Synergy H1, BioTek, USA) to measure the absorbance value at a wavelength of 450nm. Detect the proliferation of cells in different treatment groups at 0, 24, 48, 72, and 96 hours in sequence, and finally export the data for processing and analysis.

### Clone formation assay

Resuspension SHG44 and SW1088 cells from si-NC group, si-*ZNF800*–1 group, and si-*ZNF800*–2 group into single-cell suspensions, respectively. After cell counting, the cells were spread evenly in 6-well plates at a density of 1 × 10^3^ cells per well. Then, the cells were continued to be cultured under the condition of conventional medium, and the formation of obvious cell colonies was observed under the microscope. 14 days later, the medium was removed, rinsed once with PBS, and then the cells were immersed in 4% paraformaldehyde at room temperature for 30 min for fixation. The cells were rinsed once with PBS and then stained with 0.1% crystal violet solution, incubated for 30 minutes at room temperature, and then rinsed three times with distilled water. Finally, the cell culture plates were photographed, cell colonies were counted and statistically analyzed.

### Transwell assay

A cell culture plate with a Transwell chamber was prepared and 700 μl of medium containing 20% serum was added to the lower chamber. Then resuspend SHG44 and SW1088 cells from si-NC, si-*ZNF800*–1 and si-*ZNF800*–2 groups into single-cell suspension, adjust the cell density to 1 × 10^5^/ml and add 100 μL into the upper chamber of the Transwell. After 24 hours of incubation, all the culture medium was removed, washed three times with PBS, fixed the cells with 4% paraformaldehyde for 30 minutes at room temperature, washed three times with PBS, and then stained the cells with 0.1% crystal violet solution, incubated for 30 minutes at room temperature, and then rinsed three times with distilled water. The cell culture plates were photographed, and the stained cells were counted and statistically analyzed.

### Wounding healing assay

Resuspend SHG44 and SW1088 cells from si-NC group, si-*ZNF800*–1 group, and si-*ZNF800*–2 group on 6-well plates, respectively. After the cells are fully grown, discard the supernatant and wash with PBS once. Use a black marker pen to draw several reference lines on the bottom wall of a 6-well plate, then use a sterile 200 μ l gun head to scratch along the direction perpendicular to the reference line, and cut a “wound” on the adherent cells. Rinse once with PBS to remove the scratched cells. Then add serum-free culture medium to the 6-well plate, set it to 0 hours, and take photos for recording. Continue to culture for 24 hours, and compare and record cells at the same location under the microscope according to the markers. Quantitative analysis of the invasive ability of glioma cells based on their relative distance.

### Immunohistochemistry

Normal brain tissue samples collected from 3 patients with gliomas and 3 patients with epilepsy and subcutaneous tumor tissue samples from mice were used. These samples were subsequently sent to Wuhan Sevier Biotechnology Co., Ltd. for fixation, embedding, and sectioning processing. Before the experiment, the slices were baked in an oven at 50–60 °C for 30 minutes, followed by dewaxing hydration, antigen repair, zoning, inactivation of endogenous peroxidase and biotin, and serum blocking treatment. Incubate the slices with primary antibody (1:1000) overnight in a cassette, add reaction enhancer solution dropwise, and incubate with secondary antibody (1:2000) for 40 minutes. Subsequently, DAB staining solution was prepared and observed under a fluorescence microscope. Once brown protein appeared under the microscope, staining was stopped. Afterwards, counterstain with hematoxylin for 3 minutes, followed by cleaning, dehydration, drying, neutral gum sealing, and photography under a fluorescence microscope.

### Western blotting

Brain tissue samples were removed from liquid nitrogen and quickly ground to a powder; transfected cells were collected by trypsin digestion. Protease inhibitors and RIPA lysate were added to the brain tissue samples and cells and lysed on ice for 1 hour. Subsequently, the lysate was transferred to a 1.5 mL centrifuge tube and centrifuged at 12,000 × g for 30 min at 4°C, and the supernatant was collected. The protein concentration was determined by the BCA method, in which the protein sample was mixed with 5 × loading buffer at a ratio of 4:1, and the protein was fully denatured by boiling in a metal bath at 100°C for 10 min. The initial voltage of SDS-PAGE electrophoresis was set at 80 V for 30 min, and then it was adjusted to 130 V for 70 min after the bromophenol blue indicator was introduced into the separating gel. For membrane transfer, the gel and PVDF membrane (0.22μm) were placed in a transfer clamp and fixed in the transfer tank, and 250 mA constant flow was applied for 2 h. After transfer, the PVDF membrane was closed with 5% skimmed milk powder-TBST solution for 1 h at room temperature, and rinsed with TBST for 3 times (10 min each). *ZNF800* primary antibody (1:1000 dilution) and GAPDH antibody (1:2000 dilution) were added, and the membrane was incubated overnight at 4°C on a shaker. The following day TBST was washed 3 times (10 min each time) and incubated with HRP-labeled secondary antibody (1:2000 dilution) for 1 hour at room temperature on a shaker. Finally, a chemiluminescence kit was used to develop the image, and the gray values were analyzed using ImageLab 6.0 software.

### Flow cytometry

After 48 hours of cell transfection, the cell culture was collected into a 15 mL centrifuge tube and set aside. Digest the cells with trypsin, add the culture fluid collected in the previous step, collect it into a 1.5 ml Ep tube and centrifuge at 1000 g for 5 min. The supernatant was aspirated, 1 mL of pre-cooled PBS was added, the cells were resuspended and transferred to a 1.5 mL centrifuge tube and centrifuged again at 1000 g for 5 min. Subsequently, the supernatant was aspirated, 1 mL of pre-cooled 70% ethanol was added, blown to mix, and fixed at 4ºC for 24 h. The cells were centrifuged at 1000g for 5 min, the supernatant was discarded, and the cells were resuspended by adding 1 mL of pre-cooled PBS. Centrifuge again at 1000g for 5 minutes and discard the supernatant. Propidium iodide staining solution was prepared according to the instructions of the Cell Cycle and Apoptosis Assay Kit (beyotime, Shanghai, China), and 0.5 mL of Propidium iodide staining solution was added to each tube of cell samples, and the cells were incubated at 37ºC for 30 minutes away from light. Detect by flow cytometry at 488nm and import the data into Modifit for analysis.

### Subcutaneous xenograft tumor experiments in nude mice

BALB/cA-nu nude mice were purchased from Beijing Huafukang Biotechnology Co., Ltd and kept in the SPF barrier environment of the Experimental Animal Center of Henan University of Traditional Chinese Medicine. After acclimatization, 15 4-week-old male nude mice were randomly divided into three groups, SHG44-si-NC, SHG44-si-*ZNF800*–1, and SHG44-si-*ZNF800*–2. The shRNA lentivirus targeting the target gene and the empty vector control lentivirus were purchased from Servicebio. SHG44 cells were cultured to logarithmic growth phase and transfected with lentivirus according to the instructions. The cell status was assessed to be in accordance with the requirements 48 hours after transfection. After digestion of the transfected cells, the concentration was adjusted to 2 × 10⁷ cells/mL and resuspended in PBS on ice for backup. After nude mice were sterilized on the flanks, the skin was punctured and submerged for approximately 1 cm, and 100 μl of cell suspension (containing 2 × 10⁶ cells) was injected. Starting from the 7th day after inoculation, the long diameter (L) and short diameter (W) of the tumor were measured by vernier caliper every 3 days, and the volume was calculated according to V = L × W²/2. Twenty-eight days after inoculation, the animals were anesthetized with sodium pentobarbital and executed, and the complete stripped tumors were weighed and photographed. Tissue samples were stored at −80 °C for backup.

### Meta-analysis of *ZNF800* in LGG

After conducting a comprehensive search in well-known public databases such as PubMed, it was found that there were no reports on *ZNF800* in LGG. Therefore, to the best of our knowledge, this study is the first to investigate *ZNF800* expression in LGG and its impact on clinical outcomes. Numerous samples from four datasets (TCGA RNA-seq, CGGA RNA-seq, GSE43378, and GSE50025) were used to perform a meta-analysis and assess the prognostic value of *ZNF800* in patients with LGG. The GSE43378 and GSE50025 datasets included 18 and 34 LGG tissue samples from the Gene Expression Omnibus (GEO; https://www.ncbi.nlm.nih.gov/geo/) database. The Q-test showed that the heterogeneity of the four datasets was I^2^ = 49%, *P* = 0.12, and a fixed-effects model was selected and subjected to the matE program of R software (version 4.0.3).

### Immune correlation analysis of *ZNF800* in LGG

The degree of immune cell infiltration into tumor tissues is important for tumor prognosis and treatment. The Tumor IMmune Estimation Resource (TIMER; https://cistrome.shinyapps.io/timer/) database was used to study the relationship between *ZNF800* expression and immune infiltration in LGG. We studied the correlation between *ZNF800* expression and immune cell (B, CD8 + T, CD4 + T, macrophages, neutrophils, and dendritic cells) infiltration in LGG, as well as between immune cell infiltration level, survival time, and immune cell copy number change. Recently, immune checkpoint therapy led by PD-1/PD-L1 inhibitors has significantly advanced in clinical antitumor treatment, improving the survival of patients with advanced liver, lung, colorectal, and other cancers to a certain extent [18]. Subsequently, we used the Pearson correlation coefficient to explore the relationship between *ZNF800* and eight well-known immune checkpoints using R.

### Gene set enrichment analysis (GSEA)

GSEA (http://www.gsea-msigdb.org/gsea/index.jsp) is frequently used to assess gene distribution trends [19]. In this study, according to the median *ZNF800* expression, patients with LGG were categorized into high-expression and low-expression groups, and the relationship between *ZNF800* and the signaling pathway in LGG was analyzed. Results were considered significant when *P* < 0.05 and false discovery rate (FDR) < 0.25.

### Statistical analysis

All experiments were repeated three times and statistically analyzed using GraphPad Prism (8.0.1). The R software (4.0.3) was used to analyze and process the data. The chi-square test was used to analyze the relationship between *ZNF800* expression and different clinical features in patients with LGG. Cox regression models were used to analyze whether *ZNF800* expression was an independent prognostic risk factor for LGG. Kaplan–Meier analysis was used to analyze the effect of *ZNF800* expression on the survival of patients with LGG. The receiver operating characteristic (ROC) curve was used to evaluate the diagnostic value of *ZNF800*. The Pearson correlation coefficient was used to screen for genes co-expressed with *ZNF800*. *P*-values < 0.05 were considered statistically significant.

## Results

### *ZNF800* expression is significantly upregulated in LGG

**Fig 1:**
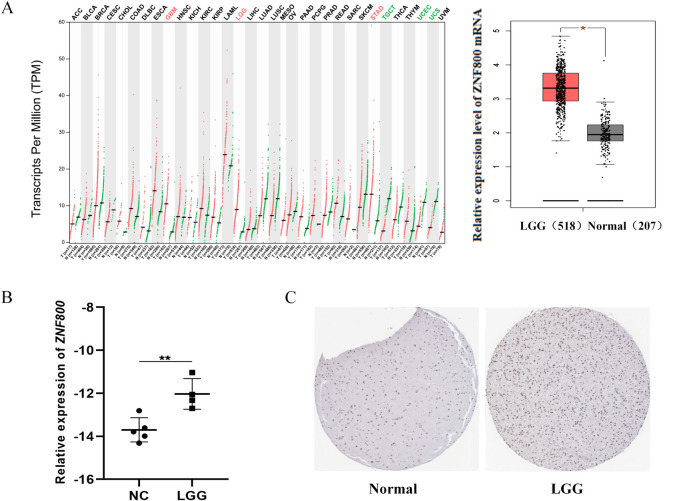
Comparison of the expression of *ZNF800* in tumor and normal tissues. (A): The expression level of *ZNF800* in LGG based on analysis of the GEPIA database. Red represents high expression, green represents low expression. (B): RT-qPCR was used to detect *ZNF800* expression in normal brain tissue and LGG tissues. (C): Representative images of IHC staining with *ZNF800* protein expression level in LGG and non-tumor tissue samples; brown represents positive staining. The experiments were repeated three times. *: p < 0.05 and **: p < 0.01; p < 0.05 is considered statistically significant.

Using the existing GEPIA database, we found that compared to 207 normal brain tissues, the transcription level of *ZNF800* was significantly higher in 518 LGG tissues (Fig 1A). To eliminate statistical bias, we validated it using RT qPCR and immunohistochemistry, and found that *ZNF800* was significantly increased in LGG tissue compared to normal brain tissue (Figs 1B and S1A). In addition, the protein expression level of *ZNF800* in the HPA database significantly increased (Fig 1C). Based on the above multidimensional verification, *ZNF800* is abnormally highly expressed in LGG, indicating that *ZNF800* is involved in the pathological progression of LGG; However, the exact mechanism is still unclear.

### High *ZNF800* expression is related to the malignant clinical characteristics of patients with LGG

**Fig 2:**
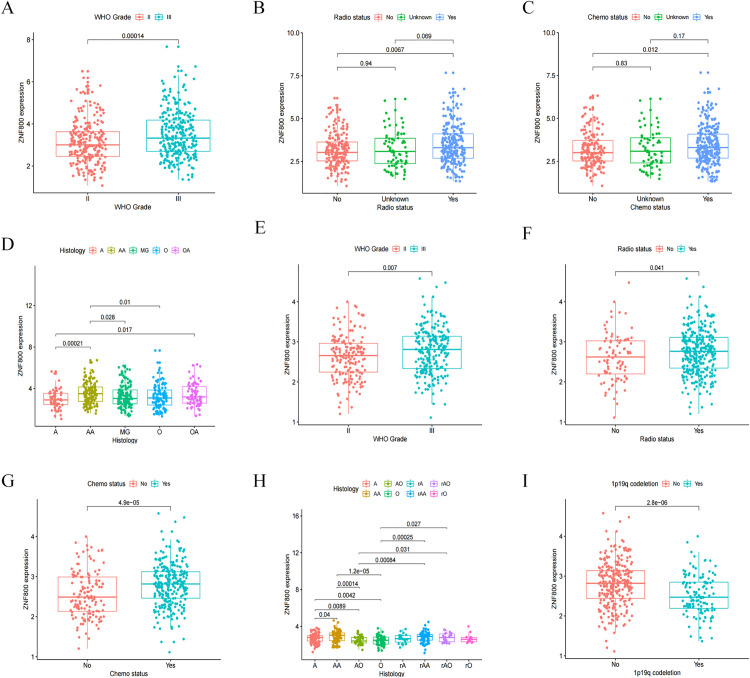
High expression of *ZNF800* is positively associated with malignant clinical characteristics of LGG patients based on TCGA RNA-seq (A,B,C,D), and CGGA RNA-seq (E,F,G,H,I) data. (A,E): High expression of *ZNF800* is positively correlated with higher WHO grade (WHO III) (P = 0.00014,P = 0.007). (B,F): LGG patients receiving radiotherapy have high expression of *ZNF800* (P = 0.0067,P = 0.041). (C,G): Compared with no treatment, a higher expression of *ZNF800* is found in LGG patients receiving chemotherapy (P = 0.012,P = 4.9e-05). (D,H): Positive correlation between *ZNF800* expression and histology of LGG patients. (I): High level of *ZNF800* is more associated with no 1p19q codeletion (P = 2.8e-0.6).

Most clinicopathological features of patients with LGG are closely associated with patient prognosis. Therefore, we investigated the relationship between *ZNF800* and the clinical features related to the prognosis of patients with LGG. First, based on the results from TCGA RNA-seq database, we found that *ZNF800* expression in patients with WHO grade III LGG, radiotherapy, and chemotherapy groups was significantly higher than that in the WHO grade II LGG, non-radiotherapy, and non-chemotherapy groups (Fig 2A, 2B and 2C). In addition, *ZNF800* expression was the highest in anaplastic astrocytoma (AA) (Fig 2D). Next, to validate the results obtained from TCGA RNA-seq database, we analyzed the CGGA RNA-seq data, which showed a high degree of agreement between the results obtained from the two databases. *ZNF800* expression was significantly and positively correlated with WHO classification, radiotherapy status and chemotherapy status, as well as *ZNF800* expression was highest in AA (Fig 2E–2H). Finally, data from the CGGA RNA-seq database showed that the expression of *ZNF800* was significantly lower in 1p19q-deficient LGG patients than in the non-1p19q-deficient group (Fig 2I). These results suggest that the abnormally high expression of *ZNF800* is closely associated with the malignant clinical features of LGG and may lead to a poor prognosis for LGG patients.

### Abnormally high *ZNF800* expression may be an independent risk indicator for poor prognosis of LGG

**Fig 3:**
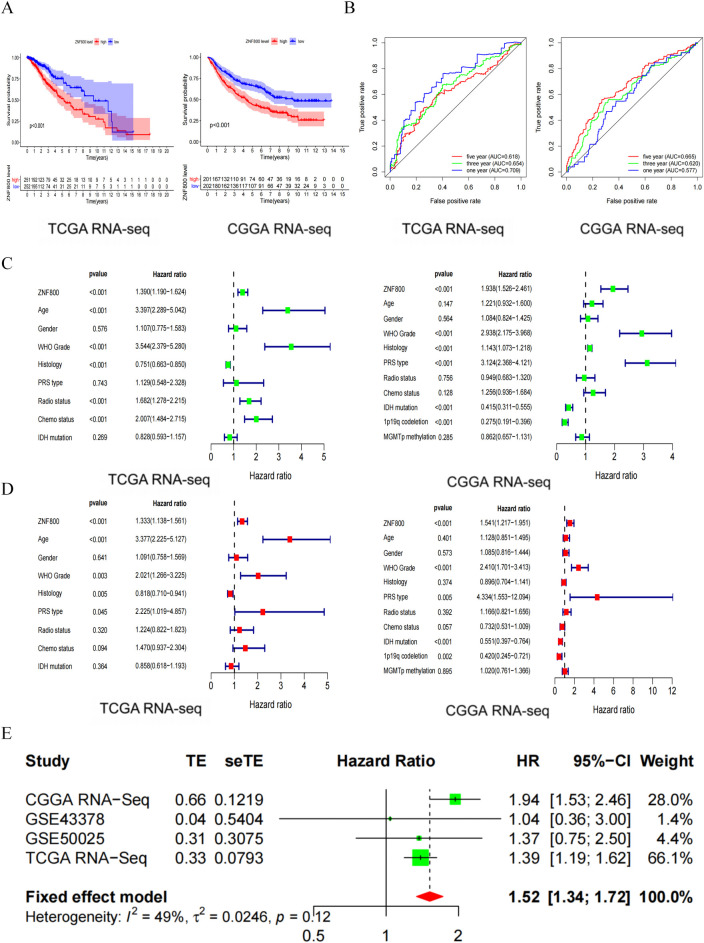
The relationship between expression of *ZNF800* and the prognosis of LGG patients based on the TCGA RNA-seq and CGGA RNA-seq data. (A): KM curves show that high expression of *ZNF800* is associated with poor overall survival in LGG patients. (P < 0.001). (B): The area under the ROC curve shows that *ZNF800* expression is related to the one-year, three-year and five-year survival rate of LGG patients (AUC > 0.5). (C): Univariate analysis was used to evaluate the risk factors of LGG patients. (D): Multivariate analysis was used to evaluate the independent risk factors of LGG patients. (E): After adding the GSE43378 dataset and GSE50025 dataset, the meta-analysis shows that the high expression of *ZNF800* is a unfavorable prognostic factor for LGG (HR = 1.52, 95%CI: [1.34; 1.72]).

To investigate whether abnormal *ZNF800* expression is associated with the prognosis of patients with LGG, the Kaplan–Meier survival curve was first used to evaluate the relationship between *ZNF800* and the overall survival (OS) of patients with LGG. Patients with higher *ZNF800* expression had significantly shorter OS than those with lower *ZNF800* expression, based on TCGA RNA-seq database. Similar results were obtained for the CGGA RNA-seq database (Fig 3A). The ROC curves based on the above two databases validated that *ZNF800* has a certain diagnosis value for patients with LGG (Fig 3B). The results of univariate and multivariate Cox regression analyses based on the above two databases showed that the hazard ratio (HR) of *ZNF800* was > 1, suggesting that high *ZNF800* expression is an independent risk indicator for prognosis of patients with LGG (Fig 3C and 3D). To verify that the highly expressed *ZNF800* is a pathogenic gene in LGG, we fused 958 LGG patient samples from four databases (CGGA RNA-seq, GSE43378, GSE50025, and TCGA RNA-seq) for a meta-analysis (Fig 3E). The pooled HR and 95% confidence interval (CI) for *ZNF800* overexpression and OS in 958 cases was 1.52 [1.34, 1.72], indicating that *ZNF800* is a pathogenic gene of LGG. These results confirm that high *ZNF800* expression is associated with shortened OS in patients, and *ZNF800* is an independent pathogenic gene of LGG. The results suggest that abnormally high *ZNF800* expression plays a key role in the poor prognosis of LGG.

### Knockdown of *ZNF800* significantly inhibited the proliferation and invasive ability of LGG cells

**Fig 4:**
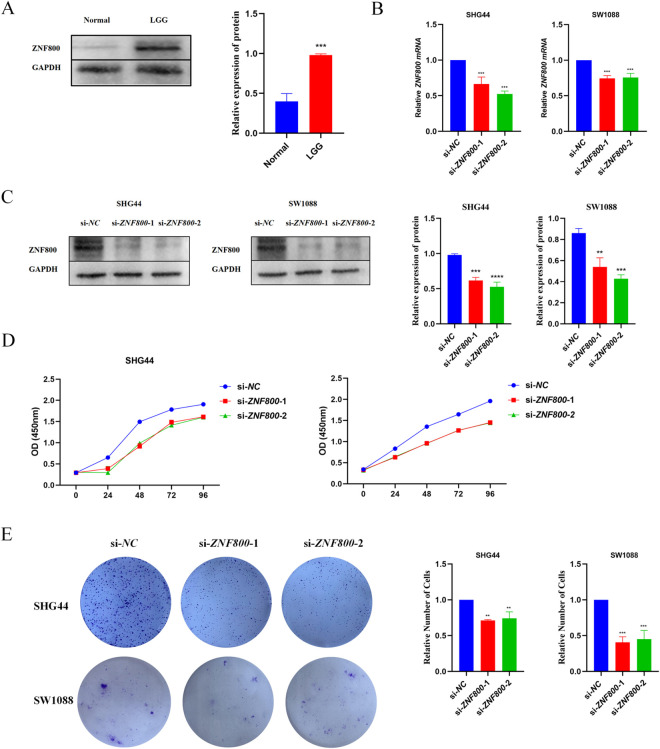
Effect of knockdown of *ZNF800* on the proliferation of glioma cell lines. (A): Western Blot showing the expression of *ZNF800* in normal and LGG tissues; (B-C): RT-qPCR and Western Blot experiments verifying the mRNA and protein expression of *ZNF800* in the si-*ZNF800*-1 group, si-*ZNF800*-2 group and si-NC group. (D): Absorbance values at different time points in the CCK8 assay; (E): Relative number of colony staining in the clone formation assay. *: *P* < 0.05; **: *P* < 0.01; ***: *P* < 0.001; ****: *P* < 0.0001.

**Fig 5:**
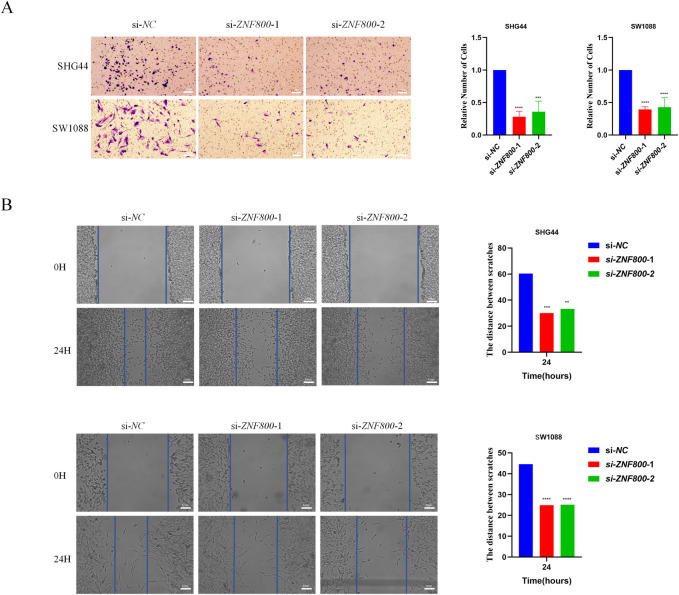
Effect of knockdown of *ZNF800* on invasion and migration of glioma cell lines. (A): Relative number of staining on the membrane at the bottom of Transwell chamber. (B): Relative migration distance of cells in wound healing assay. *: *P* < 0.05; **: *P* < 0.01; ***: *P* < 0.001; ****: *P* < 0.0001.

To elucidate the mechanism of *ZNF800* in the malignant biological behavior of LGG, we established *ZNF800* knockdown groups (si-*ZNF800*–1, si-*ZNF800*–2) and control groups (si-NC) by siRNA transfection in two LGG cell lines, namely SHG44 and SW1088. First, Western Blot confirmed that *ZNF800* protein levels were significantly higher in glioma tissues than in non-tumor brain tissues (Fig 4A). RT-qPCR and Western Blot verified that *ZNF800* was effectively knocked down at both mRNA and protein levels in both cells (Fig 4B, C). Secondly, CCK8 experiments showed that in SHG44 and SW1088 cells, compared to the si-NC group, the si-*ZNF800*–1 and si-*ZNF800*–2 groups showed a significant decrease in cell proliferation ability at 72 and 96 hours (Fig 4D). The clone formation experiment also confirmed this, with P < 0.05 (Fig 4E). Finally, the effect of *ZNF800* siRNA on the invasion of human glioma cells SHG44 and SW1088 was demonstrated through Transwell experiments and wound healing experiments. The number of stained cells on the bottom membrane of the Transwell chamber was less in the si-*ZNF800*–1 and si-*ZNF800*–2 groups than in the si-NC group, with P < 0.05 (Fig 5A). The migration ability of SHG44 and SW1088 cells significantly decreased at 24 hours after knocking down *ZNF800* (Fig 5B). The above results indicated that knockdown of *ZNF800* significantly inhibited the proliferation, invasion and migration ability of LGG cells, suggesting that *ZNF800* is a key factor driving the malignant biological behavior of LGG.

### Co-expression analysis and GSEA of *ZNF800* in LGG

**Fig 6:**
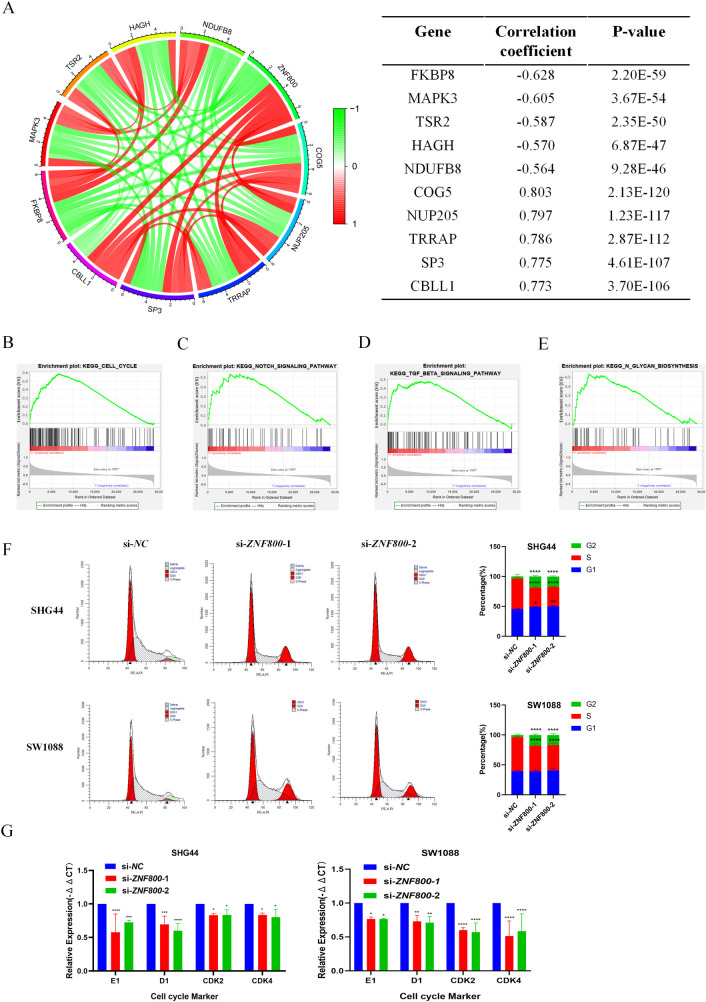
Co-expression analysis and GSEA of *ZNF800* pairs. (A): Co-expression networks show the top 10 genes that are positively and negatively correlated with *ZNF800* expression in LGG patients based on the TCGA RNA-seq database. (B-E): GSEA analysis identified mainly enriched signaling pathways with high *ZNF800* expression in LGG based on the TCGA RNA-seq database, including Cell cycle, TGF-beta signaling pathway, Notch signaling pathway, and N-Glycan biosynthesis. (F): Flow cytometry results showed that the S-phase of the cell cycle was shortened after knockdown of *ZNF800*; (G): RT-qPCR experiments showed that the mRNA levels of Cyclin E1, Cyclin D1, CDK2 and CDK4 were significantly reduced after knockdown of *ZNF800* in both SHG44 cells and SW1088 cells. *: *P* < 0.05; **: *P* < 0.01; ***: *P* < 0.001; ****: *P* < 0.0001.

To better understand the biological function of *ZNF800* in LGG, we explored the genes co-expressed with *ZNF800* in LGG using the Pearson correlation coefficient based on TCGA RNA-seq database. Co-expression analysis revealed that 2170 genes were related to *ZNF800*. We screened the 10 most related genes, of which five (COG5, NUP205, TRRAP, SP3, and CBLL1) were positively correlated with *ZNF800*, and five (FKBP8, MAPK3, TSR2, HAGH, and NDUFB8) were negatively correlated with *ZNF800* (Fig 6A). To further clarify the role of *ZNF800* in LGG, GSEA analysis showed that cell cycle, TGF-beta signaling pathway, Notch signaling pathway, and N-glycan biosynthesis were the significantly enriched signaling pathways (P < 0.05, FDR < 0.25) (Fig 6B–6E). The relevant literature search revealed that these cellular signaling pathways are involved in numerous cellular activities in cancers, such as proliferation, apoptosis, invasion, metastasis, extracellular matrix remodeling, differentiation, and immune regulation [20–23]. These results suggest that abnormally high *ZNF800* expression contributes to promoting malignant LGG transformation through multiple co-expressed synergistic genes and cell signaling pathways.

Abnormal proliferation of tumor cells is often accompanied by dysregulation of cell cycle regulation [24]. The cell cycle (G1, S, G2, M phases) is precisely regulated by Cyclin Dependent Kinase (CDK) and Cyclin [25,26]. In tumors, dysregulation of this regulatory network (e.g., Cyclin D overexpression, CDK4 amplification) drives aberrant proliferation and malignant progression [27,28]. To investigate the effect of *ZNF800* on the LGG cell cycle, flow cytometry analysis showed that the proportion of cells in S phase was significantly reduced after knockdown of *ZNF800* (Fig 6F), indicating that cell cycle progression was blocked. Further RT-qPCR experiments confirmed that knockdown resulted in significant downregulation of the mRNA levels of the key cycle regulators CyclinD1, CyclinE1, CDK2 and CDK4 (Fig 6G). These results suggest that elevated *ZNF800* expression may promote LGG cell cycle progression (especially S-phase) and proliferative capacity through the up-regulation of CyclinD1, CyclinE1, CDK2, and CDK4, thereby promoting malignant tumor progression.

### The association of *ZNF800* expression with immune cells and immune checkpoint

**Fig 7:**
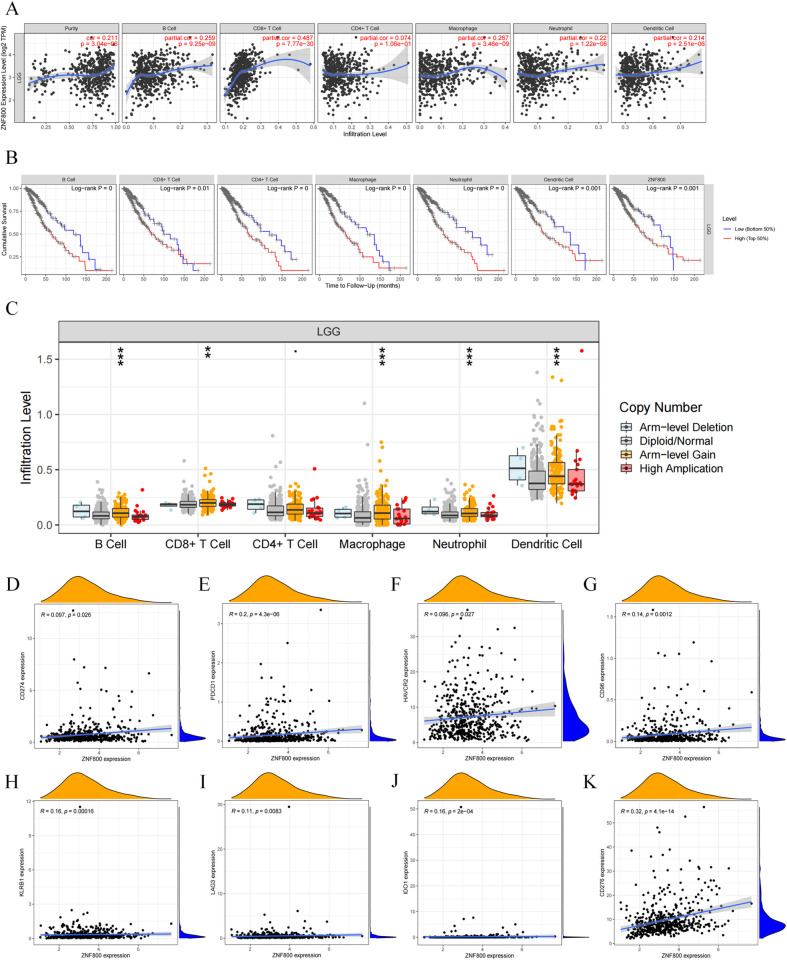
Correlation of *ZNF800* expression with immune cells and immune checkpoints. (A): *ZNF800* expression was positively correlated with five types of immune cell infiltration in LGG, including B cell, CD8 + T Cell, macrophage, neutrophil, and dendritic cell. (B): Kaplan-Meier survival analysis shows high expression of *ZNF800* and high infiltration levels of six immune cells with poor prognosis in LGG. (C): the relationship between somatic copy number alterations of *ZNF800* and immune cell infltration in LGG. (D-K): The expression of *ZNF800* was positively correlated with CD274(P = 0.026), PDCD1(P = 4.3e-06), THAVCR2(P = 0.027), CD96(P = 0.0012), KLRB1(P = 0.00016), LAG3(P = 0.0083), IDO1 (P = 2e-04) and CD276 (P = 4.1e-14).

Immune cell infiltration into the tumor microenvironment (TME) plays a key role in tumor development and influences the clinical outcomes of patients with cancer [29]. We further explored the regulatory mechanism underlying the abnormally high expression of *ZNF800* in the LGG immune microenvironment. First, *ZNF800* expression was positively correlated with the infiltration levels of five types of immune cells (B, CD8 + T, macrophages, neutrophils, and dendritic cells) using the TIMER database (Fig 7A). Second, the Kaplan–Meier survival curve showed that patients with LGG with high infiltration levels of the five immune cells in the *ZNF800* high-expression group had significantly shorter OS (Fig 7B). Third, we investigated the relationship between the copy number variation of *ZNF800* and five types of immune cell infiltration in LGG. Arm-level gain promoted a significantly increased infiltration of B cells, macrophages, neutrophils, and dendritic cells (*P *< 0.001) (Fig 7C). These results suggest that *ZNF800* influences the malignant transformation of LGG by affecting the immune microenvironment of LGG.

Finally, we explored the mechanism underlying the pathogenic function of *ZNF800* in LGG, considering that the progression of multiple malignant tumors may be related to the abnormal expression of immune checkpoint genes, and identifying new immune checkpoint will help develop new immunotherapeutic drugs [30]. Therefore, we investigated the relationship between *ZNF800* and immune checkpoints using TCGA RNA-seq database and found that *ZNF800* was positively associated with eight immune checkpoint genes, especially two well-known checkpoints, CD274 and PDCD1 encoding PD-L1 and PD-1, respectively. (Fig 7D–7K). These results strongly suggest that elucidating the role of *ZNF800* in the TME is beneficial for LGG immunotherapy.

### Knockdown of *ZNF800* inhibits LGG subcutaneous xenograft tumor growth

**Fig 8:**
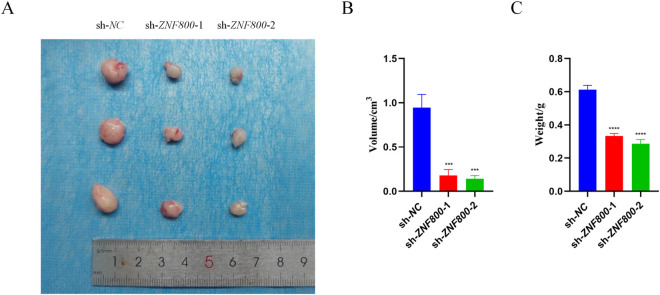
Knockdown of *ZNF800* significantly inhibits subcutaneous tumor formation in nude mice. (A): In vivo results of tumor formation in animals; (B): differential analysis of tumor volume among different groups, tumor volume was significantly reduced in the knockdown group; (C): differential analysis of tumor weight among different groups, tumor weight was significantly reduced in the knockdown group. *: *P* < 0.05; **: *P* < 0.01; ***: *P* < 0.001; ****: *P* < 0.0001.

In order to investigate the mechanism of *ZNF800*’s role in tumorigenesis, this study conducted in vivo animal model experiments and constructed a subcutaneous tumor model. A cell line (SHG44) with higher knockdown efficiency of *ZNF800* and more significant results of phenotyping experiments was selected for the study. At the same time, according to the established sequence, the lentivirus with complete package was purchased and transfection operation was carried out. SHG44 cells treated with *ZNF800* knockdown as well as control cells were inoculated subcutaneously in BALB/c nude mice, respectively. The experimental results showed that 28 days after inoculation, the tumor volume was significantly reduced in the *ZNF800* knockdown group compared to the control group (Fig 8A, B). Eventually, after the mice were euthanized, the tumor weight was measured, and it was found that there was also a significant decrease in tumor weight in the *ZNF800* knockdown group (Fig 8C). To verify the correlation between *ZNF800* and the immune checkpoint PD-L1, we performed PD-L1-specific immunohistochemical staining analysis on mouse tumor tissue sections. The results showed that in the si-NC group, PD-L1 protein showed clearly visible positive staining signals in the target cells. In contrast, in the (si-*ZNF800* group, the intensity of immunohistochemical staining of PD-L1 was significantly weakened, and the positively stained areas were markedly reduced and the staining was significantly faded. This result intuitively demonstrated that knockdown of *ZNF800* gene expression could effectively down-regulate the expression level of PD-L1 protein in tissues (S1 FigB).

## Discussion

Transcription factors are key regulators that direct genomic expression and are essential for the maintenance of transcriptional homeostasis in organisms [31,32]. Dysregulation of any transcription factor leads to abnormal activation and driving of downstream gene transcription patterns, which can lead to a series of diseases [33]. Tumors are common diseases caused by transcriptome disorders, and targeting aberrantly regulated transcription factors has become an important strategy for antitumor therapy as a convergence point of transcriptional signals [34].Glioma is a primary intracranial malignant tumor that is common and fatal. It has a poor prognosis, high heterogeneity, rapid development, and few effective treatment options. In the last ten years, numerous studies on the molecular mechanisms of LGG pathogenic genes have been conducted in full swing; among them, aberrant transcriptional activation and apoptosis inhibition play a significant role in regulating LGG cell proliferation and metastasis [35]. *ZNF800* is involved in transcriptional activation and apoptosis regulation, but the expression, regulation, biological function and mechanism of action of *ZNF800* in tumors are currently unknown [36]. To date, there have been few reports on the role of *ZNF800* in tumors. There are reports that *ZNF800* promotes the progression of lung cancer, leading to poor prognosis in cancer patients, which is similar to our research results in LGG [36].

In this study, we conducted bioinformatics analysis and experiments to verify the biological behavior of *ZNF800* in the malignant transformation of LGG through multiple public databases using 958 clinical data points. *ZNF800* expression was increased in LGG at both the mRNA and protein levels, suggesting that *ZNF800* is involved in LGG pathogenesis. *ZNF800* expression was significantly positively correlated with WHO grade, radiotherapy status, and chemotherapy status of LGG. Previous studies have suggested that with the improvement of the WHO grade, the prognosis of patients with glioma worsens [37], indicating that high *ZNF800* expression may be associated with poor prognosis in LGG. In particular, analysis based on the CGGA RNA-seq database revealed, that patients with 1p19q co-deletion LGG had significantly lower *ZNF800* expression levels than those in the non-co-deletion group, a clinically significant finding, as 1p19q co-deletion is known to be a molecular feature of oligodendrogliomas, which are usually sensitive to radiotherapy and have a better prognosis than IDH-mutant astrocytomas [38]. The low expression status of *ZNF800* in the co-deletion group may partially explain the relatively favorable clinical outcome of this subtype at the molecular level.

Translated with DeepL.com (free version)Furthermore, the ROC curve, univariate and multivariate Cox regression analyses, and Kaplan–Meier survival curve based on TCGA and CGGA RNA-seq databases showed that *ZNF800* was an independent predictor of clinical prognosis in patients with LGG, and its high expression suggests significantly reduced OS in patients with LGG. These findings suggest that as a pathogenic gene, abnormally high expression of *ZNF800* plays an important role in the malignant evolution of LGG and leads to a poor prognosis in patients with LGG. Our results are consistent with those of previous studies in which *ZNF800* expression increased in lung cancer cell lines, and the upregulation of *ZNF800* expression may contribute to poor prognosis in patients with lung cancer [36]. In the complex tumor microenvironment, the malignant behavioral actions of tumor cells, such as invasion, proliferation, and migration, are key factors contributing to tumor progression and poor prognosis. In order to further investigate the role of *ZNF800* in LGG, our research group selected glioma cell lines SHG44 and SW1088 for experiments. CCK8 assay and Clone formation assay are all closely related to cell proliferation, which fully proves that in the pathological process of LGG, the elevated expression level of *ZNF800* can significantly increase the ability of malignant proliferation of LGG cells. In addition, Transwell experiments revealed that *ZNF800* could increase the ability of low-grade glioma cell invasion, and scratch experiments proved that *ZNF800* could increase the ability of low-grade glioma cell migration. In summary, in the malignant progression of LGG *ZNF800* can increase the ability of glioma cells to proliferate, migrate, invade, and other malignant biological behaviors, which in turn leads to a poor prognosis of LGG patients. More importantly, two low-grade glioma cell lines and two siRNAs were used in this study for cell phenotyping experiments, which avoided the chance of in vitro results and made the results more convincing. The in vivo experiments confirmed the inhibitory effect of knocking down *ZNF800* on LGG growth, which is consistent with the results observed in vitro experiments, providing more clinically significant evidence for *ZNF800* as a potential therapeutic target. Overall, *ZNF800* has the potential to become a highly promising target in the treatment of LGG.

To investigate how *ZNF800* contributes to the malignant progression of LGG, GSEA analysis using TCGA database revealed that the high-expression *ZNF800* group was highly enriched in the cell cycle, TGF-beta signaling pathway, Notch signaling pathway, and N-glycan biosynthesis [39,40]. Pontin, a tumor-promoting protein, is overexpressed in gliomas, positively regulating the cell cycle, enabling cell growth, and accelerating glioma development [41]. Abnormal activation of the TGF-beta signaling pathway promotes glioma cell invasion and migration, and TGF-beta is central to immunosuppression in the TME [42,43]. In addition, the Notch signaling pathway can actively regulate the proliferation and differentiation of glioma cells by inhibiting tumor-suppressing factors, as well as promote drug tolerance in glioma [44,45]. N-glycans stimulate the invasive ability of glioma cells, while inhibition of N-glycan results in decreased glioma cell adhesion and migration abilities [46]. Thus, GSEA revealed that *ZNF800* may participate in LGG tumor cell proliferation, metastasis, and immune regulation, leading to a poor prognosis through the abnormal activation of the above signaling pathways. Flow cytometry and RT qPCR experiments have confirmed that knocking down *ZNF800* can significantly downregulate the mRNA expression levels of key cell cycle regulatory factors CyclinD1, CyclinE1, CDK2, and CDK4, thereby effectively inhibiting the normal progression of the cell cycle and suppressing tumor cell proliferation. This is highly consistent with the regulatory mode of ZNFs family members in tumor invasion, especially ZSCAN20 in the ZNFs family, which can maintain the progression of the cell cycle by regulating the expression of cell cycle related genes in hepatocellular carcinoma, thereby promoting tumor cell proliferation and invasion [47]. Furthermore, LGG has a complex pathogenesis in which multiple genes play important roles [48–50]. In oncology research, co-expression analysis can be used to screen for genes that are synergistic or antagonistic to target genes and to predict their function [51]. We screened multiple genes co-expressed with *ZNF800*, which contributes to malignant progression in LGG, through co-expression analysis. Genes positively correlated with *ZNF800* play crucial roles in LGG pathology; for example, *SP3* can indirectly induce glioma proliferation and metastasis, play a key role in the malignant biological behavior of gliomas, and lead to poor prognosis for patients [52]. Genes negatively correlated with *ZNF800*, such as MAPK3, may act as tumor suppressors that promote autophagy in glioma cells [53]. Based on previous studies and the present study, we believe that *ZNF800*, as a causative gene, together with the co-expressed genes, plays an important role in the malignant evolution of LGG.

Lin et al [54] identified *ZNF800* as a key transcription factor controlling the differentiation of enteroendocrine cells (EECs) through a CRISPR screen in human small intestinal organoids.*ZNF800* inhibits the differentiation of EECs mainly by directly binding to and repressing the expression of transcription factors, such as PAX4, SOX4, and INSM1; the binding sites of *ZNF800* are located in the promoter regions of these transcription factors and these sites have high affinity; and the absence of *ZNF800* leads to the upregulation of the expression of these transcription factors, which promotes the differentiation of EECs. The binding sites of *ZNF800* are mainly located in the promoter regions of these transcription factors, and these sites have high affinity; and the deletion of *ZNF800* leads to the up-regulation of the expression of these transcription factors, which promotes the differentiation of EEC. Among them, the expression of PAX4 was associated with the proliferation and differentiation of pancreatic cancer cells, and PAX4 may promote tumor development by regulating the cell cycle and promoting cell proliferation [55]. Similarly, in lung and breast cancers, high expression of SOX4 was associated with tumor aggressiveness and poor prognosis, and promoted tumor development by regulating the cell cycle, promoting cell proliferation and inhibiting apoptosis [56,57]. Our GSEA results also indicate that *ZNF800* is significantly associated with the cell cycle. From this, therefore, we hypothesized that in the pathological process of LGG, *ZNF800* activates the cell cycle by interacting with PAX4, SOX4, and so on, from promoting malignant tumor progression. These discussions further strengthen our previous view that *ZNF800* is a pathogenic gene in LGG patients, and its elevated expression level may result in a close correlation with the poor prognosis of patients. However, due to the small number of *ZNF800*-related studies, its specific mechanism of regulating LGG proliferation and migration, as well as the downstream/upstream genes of *ZNF800* need to be verified by our subsequent experiments.

Recently, the role of immune cell infiltration in tumor occurrence, development, diagnosis, and treatment has been the focus of researchers and clinicians [58]. Therefore, this study explored the role of *ZNF800* in the LGG immune microenvironment using the TIMER database and whether it is a target for anti-LGG immunotherapy. As the expression of *ZNF800* increased, the degree of infiltration of the five different immune cell types in the LGG immune microenvironment gradually increased, and the accompanying survival time gradually decreased. These results suggest that *ZNF800* may be involved in the formation of an LGG-suppressive immune microenvironment. Tumor cells evade immune surveillance, leading to continuous tumor progression [59]. Growing evidence suggests that immune checkpoint inhibitors developed based on immune checkpoints could play an active role in treating gliomas [60,61]. In this study, we found that *ZNF800* was positively correlated with the expression of multiple common and well-known immune checkpoints, including PD-1 and PD-L1. Therefore, we believe that *ZNF800* is a novel target for effective immunotherapy in patients with LGG.

Despite the comprehensive analysis of the effect of *ZNF800* on the pathological process of LGG in this study, there are unavoidable limitations. First, retrospective analysis has inherent limitations, and the public database platform lacked all clinical characteristics and treatment details of the patients. The exclusion and inclusion criteria were inconsistent and the processing lacked consistency due to data coming from different research centers, which could not compensate for the integrity of the sample collection process and clinical data. Therefore, we made our best efforts to compensate for this shortcoming by validating the key findings of our analysis with multiple databases and experiments. Second, the complex composition of the tumor immune microenvironment makes it difficult for a single study to fully reveal the intricate regulatory networks in this environment. Therefore, our study only discusses the relationship between *ZNF800* and immune cell infiltration and immune checkpoint sites in the LGG immune microenvironment, which needs to be further explored in the future.

## Conclusions

In this study we systematically elucidated the effects of *ZNF800* on the prognosis, underlying mechanisms and immune microenvironment of LGG patients, providing a basis for subsequent exploration of the mechanism of action of *ZNF800* in the field of oncology. This study reveals for the first time that *ZNF800* is significantly upregulated at both mRNA and protein levels in LGG patients. We confirmed that the pathogenic gene *ZNF800* is an independent risk factor for LGG patients and can be used as a potential biomarker for LGG patients.GSEA analysis revealed that highly expressed *ZNF800* may signaling pathways such as Notch Signaling Pathway, TGF-β Signaling Pathway, Cell Cycle mediating LGG malignant progression. Knockdown of *ZNF800* significantly inhibited the proliferation, invasion and migration of the two glioma cells. Comprehensive immunological analyses showed that highly expressed *ZNF800* was positively correlated with the immune checkpoint PD-L1, confirming that *ZNF800* may be a potential target for anti-tumor immunotherapy in LGG patients. These findings suggest that *ZNF800* is a novel pathogenic gene for LGG and may have potential clinical applications in the diagnosis and individualized treatment of LGG as well as in improving prognosis. More importantly, this study provides a new direction for the combination immunotherapy of LGG. However, the complexity of the tumor immune microenvironment makes it difficult for a single study to fully reveal the regulatory network, and this study only explored the relationship between *ZNF800* and immune cell infiltration and immune checkpoint sites in the LGG immune microenvironment. In the future, we plan to utilize a wider range of clinical samples and comprehensive experimental methods to further investigate the regulatory mechanism of *ZNF800* in LGG.

## Supporting information

S1 Table The detailed clinical features of LGG patients in TCGA RNA-seq. (DOCX)

S2 Table The detailed clinical features of LGG patients in CGGA RNA-seq. (DOCX)

S1 Fig Expression characteristics of *ZNF800* in LGG and its regulation of PD-L1. (A): Immunohistochemistry showed the difference of *ZNF800* expression in LGG and normal brain tissues. (B): Immunohistochemistry showing the regulatory effect of *ZNF800* knockdown on PD-L1 expression.
(TIF)

S1 File Western Blot raw data. The data information comes from figshare (https://figshare.com/articles/dataset/800_/28606970).
(ZIP)
