# Supplementary material for: Zinc finger protein 800 (ZNF800) promotes proliferation and migration of lower-grade glioma and is associated with immune infiltration
Source: PLoS One. 2025 Jul 11;20(7):e0324426. doi: 10.1371/journal.pone.0324426 (PMC12250612; doi:10.1371/journal.pone.0324426)
Supplement: S1 Table — (DOCX) [file pone.0324426.s001.docx]

**S1 Table The detailed clinical features of LGG patients in TCGA RNA-seq**

| **Covariates** | **Type** | **Total** | **Percentages (%)** |
| --- | --- | --- | --- |
| Age | <=41 | 260 | 51.69% |
|  | >41 | 243 | 48.31% |
| Gender | Female | 225 | 44.73% |
|  | Male | 278 | 55.27% |
| WHO Grade | II | 243 | 48.31% |
|  | III | 260 | 51.69% |
| Radio status | No | 187 | 37.18% |
|  | Unknown | 72 | 14.31% |
|  | Yes | 244 | 48.51% |
| Chemo status | No | 167 | 33.20% |
|  | Unknown | 66 | 13.12% |
|  | Yes | 270 | 53.68% |
| PRS type | Primary | 489 | 97.22% |
|  | Recurrent | 14 | 2.78% |
| IDH mutation status | No | 34 | 6.76% |
|  | Unknown | 378 | 75.15% |
|  | Yes | 91 | 18.09% |
| expression | High | 251 | 49.90% |
|  | Low | 252 | 50.10% |
| methylation | High | 251 | 49.90% |
|  | Low | 252 | 50.10% |
