# Supplementary material for: Zinc finger protein 800 (ZNF800) promotes proliferation and migration of lower-grade glioma and is associated with immune infiltration
Source: PLoS One. 2025 Jul 11;20(7):e0324426. doi: 10.1371/journal.pone.0324426 (PMC12250612; doi:10.1371/journal.pone.0324426)
Supplement: S2 Table — (DOCX) [file pone.0324426.s002.docx]

**S2 Table The detailed clinical features of LGG patients in CGGA RNA-seq**

| **Covariates** | **Type** | **Total** | **Percentages (%)** |
| --- | --- | --- | --- |
| PRS type | Primary | 273 | 67.74% |
|  | Recurrent | 130 | 32.26% |
| WHO Grade | II | 177 | 43.92% |
|  | III | 226 | 56.08% |
| Gender | Female | 171 | 42.43% |
|  | Male | 232 | 57.57% |
| Age | <=41 | 222 | 55.09% |
|  | >41 | 181 | 44.91% |
| Radio status | No | 88 | 21.84% |
|  | Yes | 315 | 78.16% |
| Chemo status | No | 134 | 33.25% |
|  | Yes | 269 | 66.75% |
| IDH mutation | No | 100 | 24.81% |
|  | Yes | 303 | 75.19% |
| 1p19q codeletion | No | 280 | 69.48% |
|  | Yes | 123 | 30.52% |
| MGMTp methylation | No | 165 | 40.94% |
|  | Yes | 238 | 59.06% |
