# Supplementary figures and images for: Zinc finger protein 800 (ZNF800) promotes proliferation and migration of lower-grade glioma and is associated with immune infiltration
Source: PLoS One. 2025 Jul 11;20(7):e0324426. doi: 10.1371/journal.pone.0324426 (PMC12250612; doi:10.1371/journal.pone.0324426)

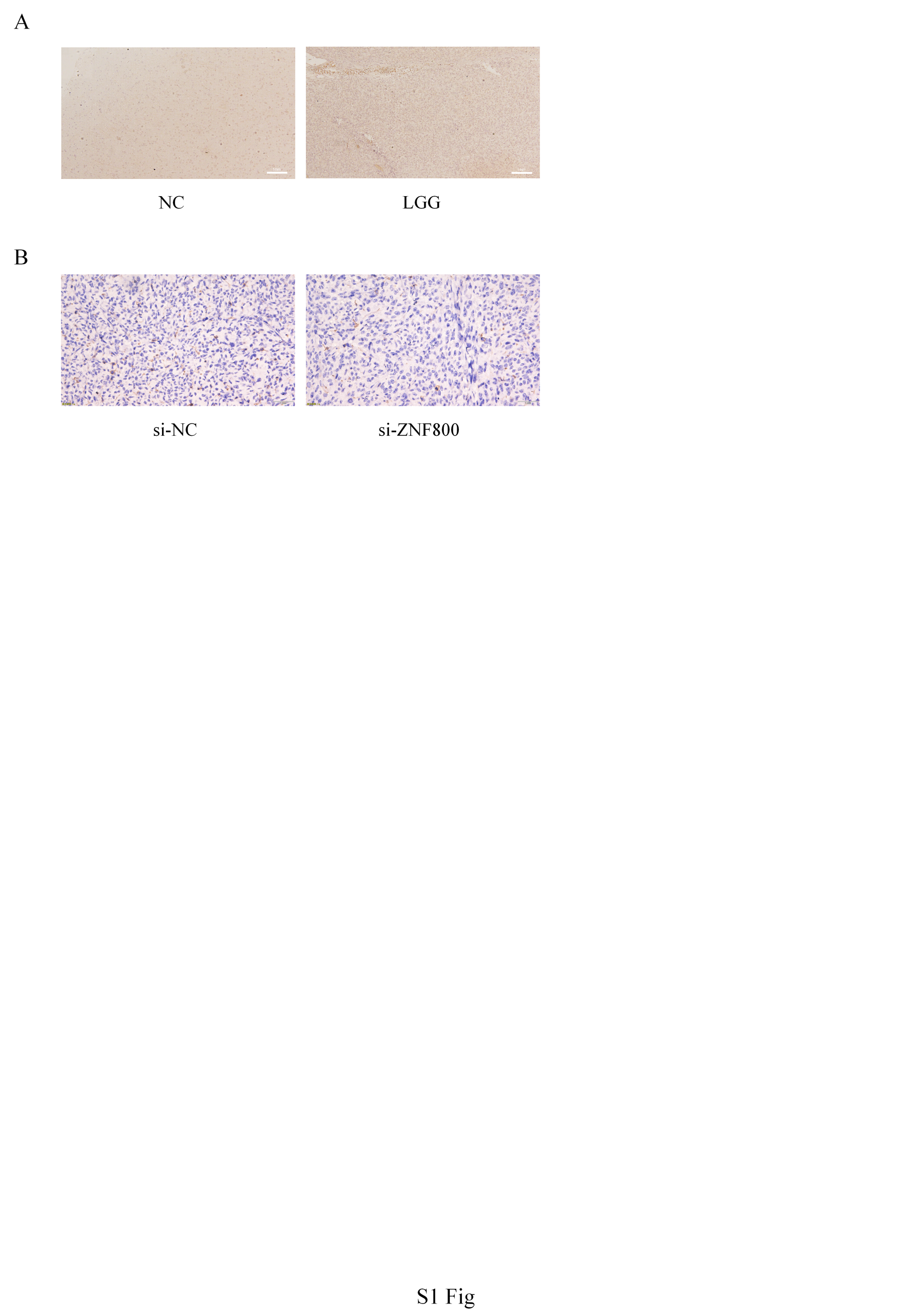

Supplement: S1 Fig — (A): Immunohistochemistry showed the difference of ZNF800 expression in LGG and normal brain tissues. (B): Immunohistochemistry showing the regulatory effect of ZNF800 knockdown on PD-L1 expression. (TIF) [file pone.0324426.s003.tif]

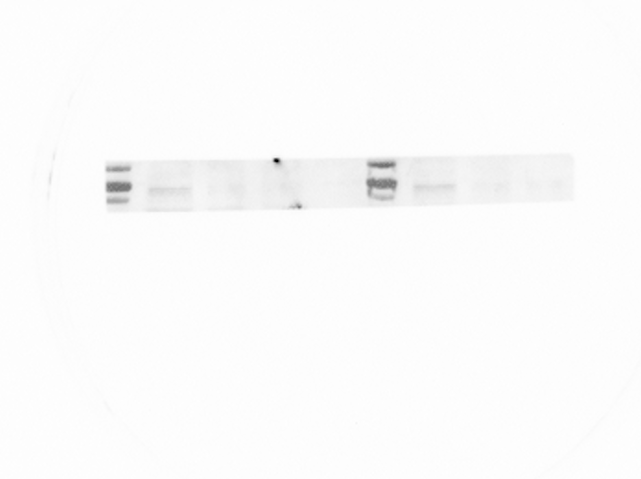

Supplement: S1 File — The data information comes from figshare (https://figshare.com/articles/dataset/800_/28606970). (ZIP) [file pone.0324426.s004.zip › Western Blot raw data/ZNF800 Knock Down/The first experiment/800.tif]

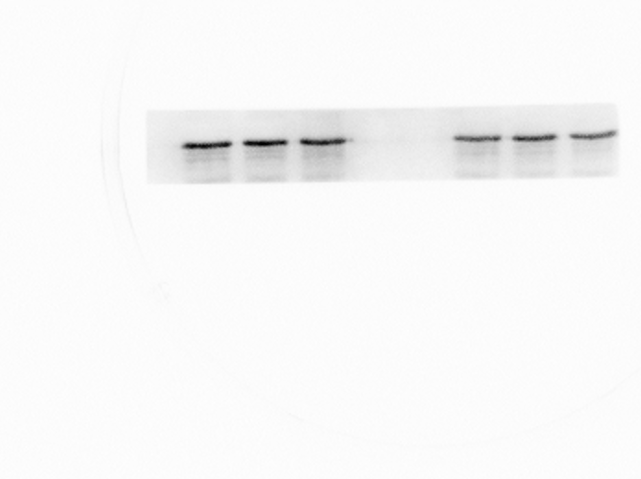

Supplement: S1 File — The data information comes from figshare (https://figshare.com/articles/dataset/800_/28606970). (ZIP) [file pone.0324426.s004.zip › Western Blot raw data/ZNF800 Knock Down/The first experiment/gap.tif]

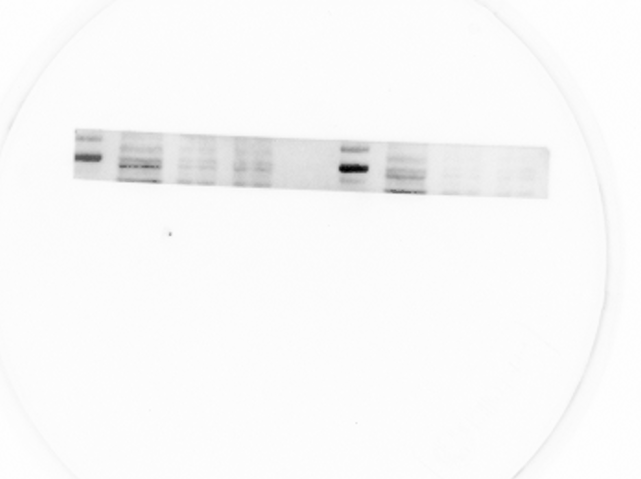

Supplement: S1 File — The data information comes from figshare (https://figshare.com/articles/dataset/800_/28606970). (ZIP) [file pone.0324426.s004.zip › Western Blot raw data/ZNF800 Knock Down/The second experiment/800-1.tif]

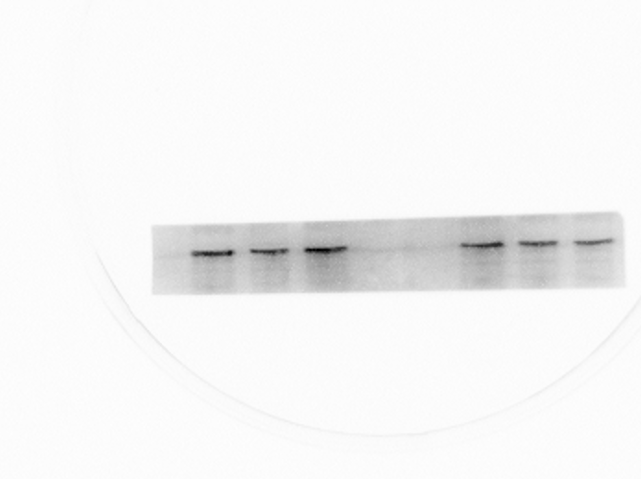

Supplement: S1 File — The data information comes from figshare (https://figshare.com/articles/dataset/800_/28606970). (ZIP) [file pone.0324426.s004.zip › Western Blot raw data/ZNF800 Knock Down/The second experiment/gap-1.tif]

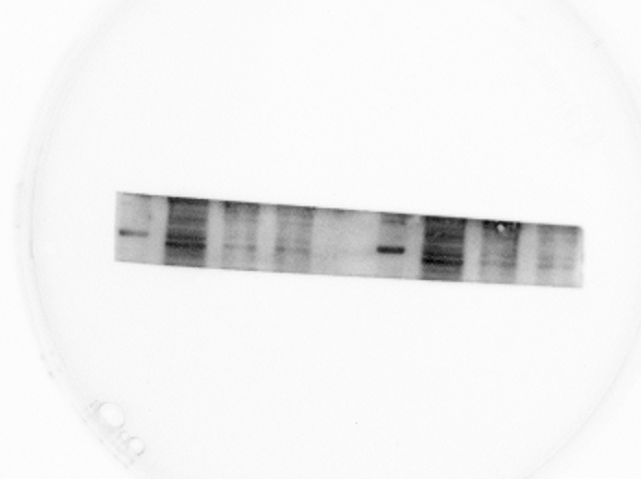

Supplement: S1 File — The data information comes from figshare (https://figshare.com/articles/dataset/800_/28606970). (ZIP) [file pone.0324426.s004.zip › Western Blot raw data/ZNF800 Knock Down/The third experiment/800-1.tif]

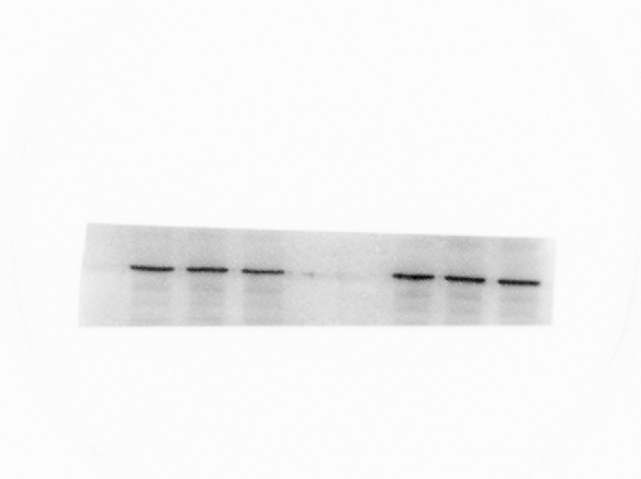

Supplement: S1 File — The data information comes from figshare (https://figshare.com/articles/dataset/800_/28606970). (ZIP) [file pone.0324426.s004.zip › Western Blot raw data/ZNF800 Knock Down/The third experiment/gap-1.tif]

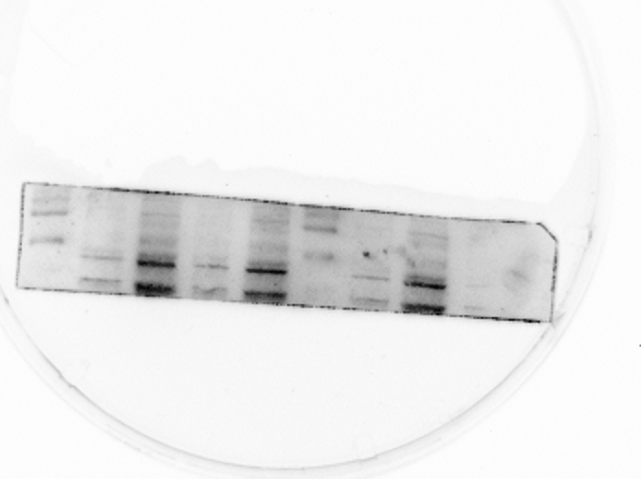

Supplement: S1 File — The data information comes from figshare (https://figshare.com/articles/dataset/800_/28606970). (ZIP) [file pone.0324426.s004.zip › Western Blot raw data/ZNF800 is expressed in organizations/800-1.tif]

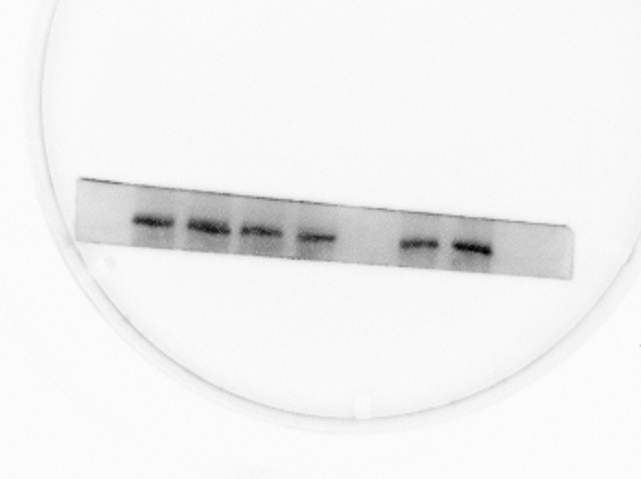

Supplement: S1 File — The data information comes from figshare (https://figshare.com/articles/dataset/800_/28606970). (ZIP) [file pone.0324426.s004.zip › Western Blot raw data/ZNF800 is expressed in organizations/gap-1.tif]
